# Telomere length is highly inherited and associated with hyperactivity-impulsivity in children with attention deficit/hyperactivity disorder

**DOI:** 10.3389/fnmol.2015.00028

**Published:** 2015-07-10

**Authors:** Danielle de Souza Costa, Daniela Valadão Freitas Rosa, Alexandre Guimarães Almeida Barros, Marco Aurélio Romano-Silva, Leandro Fernandes Malloy-Diniz, Paulo Mattos, Débora Marques de Miranda

**Affiliations:** ^1^School of Medicine, Federal University of Minas GeraisBelo Horizonte, MG, Brazil; ^2^National Institute of Science and Technology of Molecular Medicine (INCT-MM)Belo Horizonte, MG, Brazil; ^3^Department of Psychiatry, Federal University of Rio de JaneiroRio de Janeiro, Brazil; ^4^D’Or Institute for Research and Education (IDOR)Rio de Janeiro, Brazil

**Keywords:** inattention, hyperactivity-impulsivity, ADHD, biological aging, telomere length, inheritance

## Abstract

Telomere length (TL) is highly heritable, and a shorter telomere at birth may increase the risk of age-related problems. Additionally, a shorter TL may represent a biomarker of chronic stress and has been associated with psychiatric disorders. However, no study has explored whether there is an association between TL and the symptoms of one of the most common neurodevelopmental disorders in childhood: Attention Deficit/Hyperactive Disorder (ADHD). We evaluated 61 (range, 6–16 years) ADHD children and their parents between 2012 and 2014. TL was measured with a quantitative polymerase chain reaction method with telomere signal normalized to the signal from a single copy gene (*36B4*) to generate a T/S ratio. Family data was processed through a generalized estimated equations (GEE) model to determine the effect of parental TL on children TL. Inattentive and hyperactive-impulsive symptoms were also evaluated in relation to TL. For the first time, we found general heritability to be the major mechanism explaining interindividual TL variation in ADHD (father-child: 95% CI = 0.35/0.91, *p* < 0.001; mother-child: 95% CI = 0.38/0.74, *p* < 0.001). The hyperactive-impulsive dimension of ADHD was related with children’s TL (*r* = −339, *p* = 0.008) and maternal TL (*r* = −264, *p* = 0.047), but not with paternal TL (*p* > 0.05). The ADHD inattentive dimension was not significant associated with TL in this study (*p* > 0.05). TL was shown to be a potential biomarker of the ADHD symptoms burden in families affected by this neurodevelopmental disorder. However, it is crucial that future studies investigating the rate of telomere attrition in relation to psychiatric problems to consider the strong determination of TL at birth by inheritance.

## Introduction

Telomeres are DNA structures that protect a chromosome’s end from deterioration or from fusion with neighboring chromosomes, maintaining genomic stability (Blackburn and Gall, 1978). Over time, due to each cell division, the telomere ends become shorter. Telomere shortening during life is modulated by factors such as oxidative stress and adversity (Drury et al., 2012). However, telomere length (TL) at birth is highly determined by inheritance (De Meyer et al., 2011; Broer et al., 2013). Therefore, a shorter telomere at birth may increase the risk of age-related problems, and it is very important to know more about the mechanisms and consequences of TL inheritance (De Meyer et al., 2011).

TL is heritable (estimates ranging from 34 to 82%) with most of the shared variance among relatives resulting from genetic factors (Broer et al., 2013). The genetic mechanisms of telomere inheritance have been discussed in the literature, but the topic is still controversial. A study by Nawrot et al. (2004) suggested a maternal inheritance based on the X-link mechanism, resulting in a parent-of-origin effect. However, there is increasing evidence that father age at birth also influences TL (Nordfjäll et al., 2005; Vasa-Nicotera et al., 2005; Njajou et al., 2007; Atzmon et al., 2010; Chiang et al., 2010; Broer et al., 2013). By combining data from six different populations, Broer et al. (2013) recently showed a stronger mother–offspring correlation than father–offspring correlation. Although this study had a sample size larger than all previous published samples combined, Eisenberg (2014) showed no significant difference between mother–offspring and father–offspring TL after combining correlation coefficients across all previous human studies, including estimates of father–offspring and mother–offspring correlations of blood TL. Eisenberg points to the high heterogeneity across studies on both genetic and environmental determinants of TL and to the necessity of more examination of specific parental effects on this matter.

Shorter TL has been associated with psychiatric disorders such as Bipolar type II disorder and other mood disorders marked by depressive symptoms (Simon et al., 2006; Lung et al., 2007; Hartmann et al., 2010). Additionally, shorter TL was related to Posttraumatic Stress Disorder (O’Donovan et al., 2011) and schizophrenia (Kao et al., 2008). However, there is evidence suggesting that shorter TL may not be an intrinsic feature of mental disorders (Savolainen et al., 2012; Shaffer et al., 2012) but a biomarker of the effect of experienced adversity (Drury et al., 2012) and an impoverishment of psychological resources to address adverse situations (Zalli et al., 2014). In fact, the dynamics of biological sensitivity to context is calibrated by developmental experience in addition to heritable variation (Boyce and Ellis, 2005). Attention Deficit/Hyperactive Disorder (ADHD) represents a group of high genetic vulnerability, with *dopaminergic* genes among the most important components in the etiology of the disorder (Gizer et al., 2009). Because genetic vulnerability is important to stress reactivity by altering the magnitude of the association between social environment, children’s well-being and TL (Mitchell et al., 2014), ADHD is of particular interest to study the putative association between TL and psychiatric disorders. However, according to our knowledge, no empirical research has addressed TL and ADHD.

ADHD is substantially inherited (more than 70% of heritability) and is characterized by two complex behavioral traits: inattention and hyperactivity-impulsivity (HI; Doyle et al., 2005). ADHD is a major health condition and should not be viewed only as a disorder affecting children’s behavior and learning (Barbaresi et al., 2013). The cumulative burden of ADHD through the lifespan is considerable, including mortality (in addition to increased risk for early death due to suicide), social adversity [(risk for later criminal behavior and increased rates (~84%) of other mental health problems (e.g., substance abuse disorder, anxiety disorder, mood disorder, personality disorders, and disruptive behavior disorders; Barkley et al., 2008; Barbaresi et al., 2013)]. Compared with typically developing peers, ADHD children have additional social disadvantages (lower income, lower educational attainment, higher rates of school dropout; Biederman and Faraone, 2005).

In this paper, we investigated the association between parent–offspring TL in ADHD. We further sought to investigate whether child inattentive and/or hyperactive-impulsive symptoms were related to child or parental TL. Age, parental education and economic class were also evaluated in relation to TL.

## Materials and Methods

### Participants

Subjects were 61 (range, 6–16 years) ADHD children and their parents from two Southeast Brazilian Capitals: Rio de Janeiro (*n* = 32) and Belo Horizonte (*n* = 29). Thirty-three children had both parents enrolled in the study, four children had only fathers, and 24 children only mothers. Overall, 37 fathers (range, 30–55 years at the time of assessment) and 57 (range, 25–62 years at the time of assessment) mothers were enrolled. The Ethics Committees from the Federal University of Rio de Janeiro and the Federal University of Minas Gerais approved the study. All subjects provided written informed consent before enrollment. Families from both cities had spontaneously sought the psychiatry services of the University’s Hospitals concerned by children’s inattentive and/or externalizing behavior. At the time of the diagnostic interview, parents and children were invited to participate in an inter-university consortium for ADHD research. Those families who consented biological data extraction were included in this study. All family assessment was performed between 2012 and 2014.

Parents underwent a semi-structured psychiatric diagnostic interview with the Brazilian version of the K-SADS-PL (Brasil, 2003), and the diagnosis of ADHD was established in accordance with the DSM-IV criteria [American Psychiatric Association (APA), 1994]. Thirty-three (54%) children were diagnosed with the inattentive subtype (ADHD-I), five (8%) with the hyperactive subtype (ADHD-H), and 23 (38%) with the combined subtype (ADHD-C). One child had both parents reporting as being diagnosed with ADHD, and seven other children had at least one parent reporting as being diagnosed with ADHD (three fathers and four mothers).

### Measures

#### Relative Telomere Length

Peripheral blood samples were collected in tube containing ethylenediaminetetraacetic acid (EDTA), followed by DNA extraction with high salt method (Lahiri and Nurnberger, 1991). DNA was quantified Using NanoDrop Spectrophotometer and diluted to 75 ng in 96 well plates. TL was measured using a relative quantification method described previously (Drury et al., 2012). Telomere repeat length (T) to a single-copy reference gene (36B4) (S) ratio (T/S) reflects the size of telomere for each sample. The *36B4* gene, which encodes acidic ribosomal phosphoprotein PO, is located on chromosome 12 (Boulay et al., 1999). The telomere reaction proceed for one cycle at 95°C for 10 min, followed by 18 cycles at 95°C for 15 s and 54°C for 2 min and primers used were Tel-1 primer (GGT TTT TGA GGG TGA GGG TGA GGG TGA GGG TGA GGG T) and Tel-2 primer (TCC CGA CTA TCC CTA TCC CTA TCC CTA TCC CTA TCC CTA). The 36B4 reaction proceeded for one cycle at 95°C for 10 min, followed by 30 cycles at 95°C for 15 s and 58°C for 1 min 10 s and primers used were 36B4u (CAG CAA GTG GGA AGG TGT AAT CC), 36B4d (CCC ATT CTA TCA TCA ACG GGT ACA A). Primers specifically designed to amplify telomeric hexamer repeats, without generating primer dimer-derived products, were used as described previously (Cawthon, 2002). Briefly, primers were planned to allow DNA polymerase to extend from its 3′-ends only when hybridized with genomic telomeric regions. Additionally, modifications on the primers 5′-ends and a mismatch strategically placed inside each one, block DNA synthesis starting in the middle of amplification products or from primer dimers. All samples for both the telomere and single-copy gene (36B4) reactions were performed in triplicate on different plates in the same well position. Interplate coefficients of variations for the threshold cycle (Ct) values were below one percent for both the telomere and single gene reaction Cycle thresholds for each telomere and control gene 36B4 PCR reactions were calculated using the ABI software algorithm. Considering the exponential kinetics of the PCR reaction, this ratio may be expressed as the following equation: 2-ΔCt where ΔCt = Cttelomere-Ct control gene 36B4 of sample *n*. For PCR reactions, PlatinumTaq (Invitrogen) was used and amplicon formation was monitored using SYBR-Green fluorescent dye (Invitrogen). All PCR reactions and fluorescence collection were carried out in an ABI-7500 real-time PCR machine (ABI).

#### Assessment of Socioeconomic Status

Socioeconomic status was assessed using the Brazilian Criteria of Economic Classification (CCEB) according to the criteria established by the Brazilian Research Enterprises Association (Associação Brasileira de Empresas de Pesquisa (ABEP), 2008). The CCEB estimates the purchasing power of families living in urban areas. It includes nine items that measure the available resources at home and one item that considers the education level of the householder, resulting in a scale ranging from 0–46 points and classification into eight economic classes. These economic classes are divided into three classes that are more heterogeneous: “high” (A and B classes; median monthly household income of U$1048 to U$5308), “middle” (C class; median monthly household income of U$418 to U$629), and “low” (D and E classes; median monthly household income of U$194 to U$291).

#### ADHD Symptoms

As stated before, parents underwent a semi-structured psychiatric diagnostic interview with the Brazilian version of the K-SADS-PL (Brasil, 2003) and current Inattentive and Hyperactive-Impulsive symptoms were registered. The sum of Inattentive and Hyperactive-Impulsive symptoms can vary from 0–9 for each dimension.

#### Statistics

Parental TL effect on children’s TL (family data) was calculated using generalized estimated equations (GEE) with a linear regression model, robust estimators, and exchangeable structure for working correlation matrices. The dependent variable was children’s TL. Independent variables were maternal and paternal TL. Effects of maternal and paternal TL on children’s TL were controlled for parental age and education. For the GEE model, variables were transformed into z-scores using the means and SDs of the sample to facilitate effect sizes interpretation.

Bivariate relationships between ADHD symptoms (i.e., Inattention and HI) and TL (relative T/S ratio) were assessed through Spearman correlations. Due to the small sample size associations between variables were investigated by a resampling approach (*bootstrapping k* = 5000). In addition, a general linear model was performed to test the effect of socioeconomic class on TL (relative T/S ratio). We conducted all statistical procedures in SPSS 20.0.

## Results

Children were 48 (79%) boys and 13 (21%) girls from 6 to 16 years old (11.03 ± 2.44, median = 11) with average inattentive level of 7.54 symptoms (SD = 1.61, median = 8) and with average hyperactive-impulsive level of 5.33 symptoms (SD = 2.45, median = 5). Fathers had a mean age of 40.92 (SD = 6.85, median = 39) and 13.10 (SD = 3.02, median = 15) average level of education. The mean age of mothers was 41.00 (SD = 7.88, median = 44) and the mean education level was 11.86 (SD = 2.81, median = 13). Participants’ mean relative TL (T/S ratio) were 1173.02 (SD = 806.00, median = 1764.58), 1379.51 (SD = 766.06, median = 1357.86) and 1088.93 (SD = 480.50, median = 1230.51) for children, fathers and mothers, respectively. In this study, 16 (26%) children were from the high class (all at the B stratum), 39 (64%) were from the middle class, and six (10%) from the low class. There was no significant difference in TL among socioeconomic classes for children (*F*_(2,61)_ = 0.648, *p* = 0.530), fathers (*F*_(2,37)_ = 0.522, *p* = 0.599), or mothers (*F*_(2,57)_ = 0.602, *p* = 0.554).

As reported in Table 1, controlled for parental age and education, paternal (*B* = 0.63, CI = 0.35/0.91, *p* < 0.001) and maternal (*B* = 0.56, CI = 0.38/0.74, *p* < 0.001) relative TL were positively and highly related to TL in offspring. Maternal age (*B* = 0.20, CI = −0.15/0.28, *p* = 0.008) and education (*B* = 0.25, CI = 0.10/0.40, *p* < 0.001) were the only demographic variables related to children’s TL.

**Table 1:** **Effects of parental telomere length and sociodemographic variables on ADHD children telomere length (T/S ratio)**.

Outcome	Predictor	*B*	95% CI	*p-value*
TL_child_	TL__father_	0.63	0.35/0.91	0.001
	TL__mother_	0.56	0.38/0.74	0.001
	Age__child_	0.06	−0.16/0.27	0.606
	Age__father_	−0.01	−0.18/0.16	0.895
	Age__mother_	0.20	0.05/0.35	0.008
	Education__father_	0.07	−0.15/0.28	0.542
	Education__mother_	0.25	0.10/0.40	0.001

*Note: TL, Telomere length; CI, Confidence Interval*.

**Figure 1:**
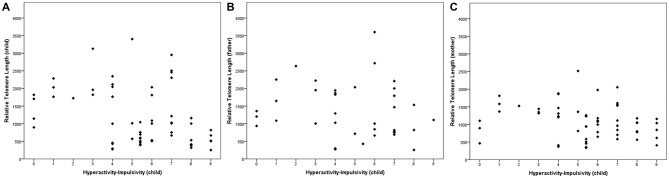
**Telomere length (TL) in relation to child’s hyperactivity-impulsivity (HI). (A)** The children’s HI level was associated with their TL (*r* = −0.339, *p* = 0.008). In parents, **(B)** we found a non-significant correlation between HI and paternal TL (*r* = −0.234, *p* = 0.164) and a **(C)** significant association between HI and maternal TL (*r* = −0.264, *p* = 0.047). Correlations were performed with a bootstrapping strategy *(k = 5000)*.

Table 2 shows the association between inattentive and hyperactive-impulsive symptoms and TL. We observed that higher levels of HI were associated with shorter relative TL in ADHD children (*r* = −0.34, *p* = 0.008) and in their mothers (*r* = −0.26, *p* = 0.047), but there was no significant association between children’s HI and fathers’ TL (*p* > 0.05). Inattentive symptoms were not related to TL in this study (*p* > 0.05). The association between children’s hyperactive-impulsive symptoms and TL is graphically shown in Figure 1.

**Table 2:** **Association between ADHD symptoms and relative telomere length (T/S ratio) (*k* = 5000)**.

	Inattention__child_				Hyperactivity-impulsivity__child_			
	*r*	*p-value*	SE_boot_	CI	*r*	*p-value*	SE_boot_	CI
TL__child_ (*N* = 61)	0.144	0.268	0.126	−0.12/0.37	−0.339*	0.008	0.118	−0.55/−0.08
TL__father_ (*N* = 37)	0.107	0.527	0.158	−0.22/0.40	−0.234	0.164	0.142	−0.50/0.06
TL__mother_ (*N* = 57)	0.177	0.189	0.129	−0.09/0.42	−0.264*	0.047	0.126	−0.50/−0.01

Note: TL, Telomere length; SE_boot_, Standard Error; CI, Confidence Interval. *Significant at p < 0.05.

## Discussion

In this data set, correlates parameter estimates from GEE model showed high effects of paternal TL and maternal TL on ADHD children TL, suggesting the majority of variance in ADHD children’s TL to be accounted for by relatedness. Additionally, we found the hyperactive-impulsive dimension of ADHD to be related with children’s TL and maternal TL, but not with paternal TL. The ADHD inattentive dimension was not significant associated with TL in this study. These results offer further support for the hypothesis that TL (or alterations in cellular aging) may be a pathway by which individual experiences during development get “under the skin” in high genetic sensitivity groups such as ADHD and impact health outcomes (Boyce and Ellis, 2005; Drury et al., 2012; Mitchell et al., 2014).

Previous results have associated TL with early childhood adverse experiences or adverse environment (Kananen et al., 2010; Tyrka et al., 2010; Drury et al., 2012; Mitchell et al., 2014) and with psychiatric conditions in childhood such as autism (Li et al., 2014). Notwithstanding, this is the first study extending these findings to include HI in childhood as a factor related to TL, at least in an ADHD context. An association between ADHD symptoms and TL is expected if the individual rate of TL attrition is established epigenetically, and early childhood is a critical period for the interaction of stress, cellular aging, and neurodevelopment (Cameron and Demerath, 2002; Mitchell et al., 2014). Some previous studies have shown the presence of adversity in ADHD (Biederman et al., 2002; Mulligan et al., 2013). If inattention is classically associated with academic failure, HI seems to be a dimension that is more associated with the development of oppositional defiant disorder and lack of support at home (Counts et al., 2005; Mulligan et al., 2013). A study by Mulligan et al. (2013) showed that among ADHD children aged less than 10 years, a poor physical home environment or less learning opportunities are associated with more hyperactive-impulsive symptoms than the children with a better environment. Pingault et al. (2011) found that hyperactivity was strongly correlated with physical aggression and opposition but not with final graduation degree. Hyperactive symptoms are most common in earlier ages and decreases in most ADHD children after 8 years old (Larsson et al., 2011; Pingault et al., 2011), which suggests that HI may begin to be translated into related life adversities too early in ADHD. It is important to note that executive function deficits, often described in ADHD children, were related to impairment in coping with stressful situations (Johnson, 2012) and heightening the impact of adversity in biological systems (Tomalski and Johnson, 2010). This finding stresses the importance of precocious treatment and development of coping abilities in ADHD. By succeeding with treatment and decreasing symptoms, we would expect a decrease in psychosocial stress and smaller shortening of telomeres. Further knowledge of the understanding of the effects of environmental and other epigenetic factors on the association between ADHD and TL is needed. However, reducing hyperactive-impulsive behaviors in early childhood (telomere attrition is more rapid in the first decade of life; Frenck et al., 1998) might be important to decrease the influence of stressful events and adversity on ADHD on a biological level through telomere maintenance, as it occurs in other therapeutic interventions designed to mitigate adverse effects of psychosocial stress (Price et al., 2013).

The influence of maternal age and education on children’s TL is also worth noting. Paternal age has been shown to be a main predictor of offspring TL (Kimura et al., 2008; Broer et al., 2013), yet only maternal age was positively associated with TL in our ADHD sample. Unlike TL in sperm which increases with age, TL in oocytes decreases with age (Wright et al., 1996, 2001; Liu et al., 2007). Therefore, a positive association between maternal age and larger TL in offspring seems implausible and a sampling bias is likely to explain our results, but further studies are needed to verify possible specificities of ADHD TL determinants. Maternal education is predictive of many indicators of child health, such as children minimal growth in regions of slums (Lartey et al., 2000) or prevention of obesity in developed countries (Matthiessen et al., 2014). In children with social disadvantages, maternal education is a predictive factor of telomere shortening (Mitchell et al., 2014). If TL is considered a biomarker of adversity, mother education can be understood as an epigenetic factor that decreases the effect of telomere shortening and possibly its clinical consequences.

In our sample, mothers were likely to be more affected by the ADHD children’s symptoms than fathers were. Being the caretaker of individuals with chronic illnesses, including caregivers of children with disabilities, has been associated with shortening of TL (Damjanovic et al., 2007; Chen et al., 2015). Mothers of ADHD children usually have higher levels of depressive disorders (Margari et al., 2013) and report more stress in parenting their children than fathers (van Steijn et al., 2014). Because parenting stress is related to negative parent-child interactions (Hastings, 2002; Harpin, 2005) parent-mediated interventions should include maternal stress reduction (Herring et al., 2006). In ADHD, genetic sensitive adolescents who experienced less responsiveness and stimulating early maternal care exhibit more symptoms of ADHD (Nikitopoulos et al., 2014), but positive parenting interaction predicts fewer future conduct problems (Chronis et al., 2007).

Finally, we would like to stress the genetic component of TL. In ADHD populations, similarities between relatives are almost completely due to genetic influence. Shared environmental influences also generally result in similarities between relatives and may moderately contribute to several developmental disorders, but ADHD exceptionally almost does not suffer shared environmental influences (Burt, 2009, 2010; Burt et al., 2012). Because TL is highly heritable, a reduced TL, at a specific time point, does not indicate a greater rate of TL decrease over time due to epigenetically established regulation of TL attrition. In a cross-sectional study design may be important to control for parental TL to ensure that a psychiatric population has a shorter TL, if compared to a healthy one, due to more psychosocial stress during life and not due to inherited shorter TL. Zhang et al. (2014), for example, also suggested that age effects were not detectable in a Posttraumatic Stress Disorder group because they already had shorter telomeres.

We should note limitations of the current study. Differences in sample composition or TL assessments may lead to divergent estimates (Horn et al., 2010), and this study is the first to investigate an ADHD population, which increases the need for studies with larger samples and different methods to strengthen our results. An important limitation is the lack of evaluation of parental psychopathology, which might contribute to the observed effect on TL. Additionally, children evaluated in this study had a mean age of 11 years old; therefore, a possible association of the inattentive symptoms with TL could not be observed in this study because stressful events related to inattention, such as chronic academic failure (Willcutt et al., 2012), may be found on TL later in life. Specific home environment confounders such as violence, child previous exposure to institutional care, marital status of parents and number of partners of the mothers until the time of children’s TL assessment were not investigated. Moreover, despite of clinical ADHD diagnosis, children were not characterized for any specific gene variants to determine genetic vulnerability.

In this study, for the first time, we found general heritability to be the major mechanism explaining interindividual TL variation in ADHD. Moreover, we demonstrated that the association between HI and TL is detectable during childhood. Our results also suggest a relationship between child hyperactive-impulsive symptoms and maternal TL. Additionally, the higher the maternal education the longer the ADHD children TL. In conclusion, TL could be a useful biomarker of the burden of psychopathology in families affected by neurodevelopmental disorders. However, it is crucial that future studies investigating the rate of telomere attrition in relation to psychiatric problems to consider the strong determination of TL at birth by inheritance.

## Conflict of Interest Statement

The authors declare that the research was conducted in the absence of any commercial or financial relationships that could be construed as a potential conflict of interest.
